# Determination of Flunixin Meglumine in Veterinary Pharmaceutical Formulations by Capillary Electrophoresis With Capacitively Coupled Contactless Conductivity Detection

**DOI:** 10.1002/elps.70114

**Published:** 2026-05-23

**Authors:** Letícia M. de Araujo, José A. Fracassi da Silva, Dosil P. de Jesus

**Affiliations:** ^1^ Institute of Chemistry Universidade Estadual de Campinas (UNICAMP) Campinas São Paulo Brazil; ^2^ National Institute of Science and Technology of Bioanalytics Lauro Kubota (INCTBio‐LK) Institute of Chemistry Universidade Estadual de Campinas (UNICAMP) Campinas São Paulo Brazil

**Keywords:** electrochemical detection, electromigration, pharmaceutical analysis, veterinary drugs

## Abstract

This work describes a novel, simple, fast, reliable, and environmentally friendly analytical method for the determination of the anti‐inflammatory and analgesic drug flunixin meglumine (FM) in veterinary pharmaceutical formulations. The method uses capillary electrophoresis with capacitively coupled contactless conductivity detection (CE‐C^4^D) to quantify meglumine (*N*‐methylglucamine), the counterion of flunixin in FM. This approach allows the use of the less expensive and more readily available meglumine, rather than FM or flunixin, as the standard reagent. Lithium ions were used as an internal standard, and the migration time of the meglumine was 2.2 min using a capillary with a total length of 50 cm (42 cm effective) and 50 µm (i.d.), filled with a background electrolyte (BGE) composed of 2‐(*N*‐morpholino)ethanesulfonic acid (MES) and histidine (His), each at 20 mmol L^−1^. Good linearity was attained (*R* ^2^ = 0.992) in a concentration range of 100–2500 µg mL^−1^. The limits of detection (LOD) and quantification (LOQ) were calculated as 22.7 and 75.6 µg mL^−1^, respectively. The accuracy of the method was assessed using recovery tests at three concentration levels, resulting in recoveries ranging from 89% to 104%. The intra‐ and inter‐day precisions ranged from 2.3% to 4.2%. The proposed CE‐C^4^D was successfully applied to determine FM in commercial veterinary formulations.

## Graphical abstract

A CE‐C^4^D method was developed for the indirect determination of flunixin meglumine (FM) by quantifying meglumine and using lithium ions as an internal standard. The method allows the use of a low‐cost standard reagent, providing suitable analytical performance for the determination of FM in veterinary pharmaceutical formulations.

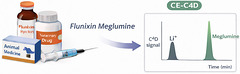

## 1 Introduction

**FIGURE 1:**
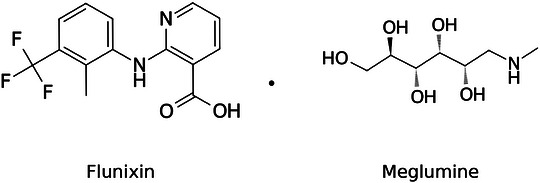
Chemical structure of flunixin meglumine.

Flunixin meglumine (FM) is a nonsteroidal anti‐inflammatory drug (NSAID) and analgesic widely used in veterinary medicine, particularly when administered intravenously [1]. FM is a salt (Figure 1) between flunixin and meglumine (*N*‐methylglucamine), and although the pharmacological activity is related to the flunixin, this association increases its aqueous solubility and makes it suitable for pharmaceutical formulations. FM acts by inhibiting cyclooxygenase, thereby reducing prostaglandin synthesis and modulating inflammatory and pain responses. In equines, FM is commonly used to relieve visceral pain, musculoskeletal inflammation, and endotoxemia [1, 2]. In cattle, FM is frequently used to treat respiratory diseases, mastitis, and other inflammatory conditions [3]. Additionally, other animals, such as swine, sheep, and goats, can benefit from the pharmacological properties of FM [4].

Determination of FM in pharmaceutical formulations is essential for quality control, stability studies, and regulatory compliance. The most used analytical methods for the determination of FM are based on high‐performance liquid chromatography (HPLC) [5, 6, 7, 8, 9, 10, 11, 12]. Nevertheless, fluorometric [13], spectrophotometric [14], and electrochemical [15, 16] methods are also reported. Although these methods provide suitable sensitivity and selectivity, most require expensive instruments, long analysis times, and significant amounts of organic solvents. Thus, the development of rapid, cost‐effective, and straightforward alternative methods for FM determination is welcome. In this context, capillary electrophoresis (CE) can be a useful analytical method for this drug because it is suitable for ionic compounds, offers high separation efficiency, short analysis time, and minimal sample and reagent consumption. Thus, minimizing chemical residues and using aqueous solutions makes CE compatible with green analytical chemistry principles. Additionally, CE can be used with capacitively coupled contactless conductivity detection (C^4^D), which relies on conductivity measurements without contact between the electrodes and the background electrolyte (BGE) within the capillary [17, 18, 19]. Capillary electrophoresis with capacitively coupled contactless conductivity detection (CE‐C^4^D) is suitable for quantifying compounds with low or no UV–vis absorption, and it has been used to quantify several compounds in various matrices [20, 21, 22]. Despite these advantages, no reports were found in the literature using CE‐C^4^D for the determination of FM, and only one work used CE with UV–vis detection for this purpose [23].

As FM is a 1:1 salt, quantifying meglumine rather than its counterion flunixin provides an interesting strategy for FM determination. Meglumine is ionizable and shows low UV–vis absorption, making CE‐C^4^D a suitable option. Additionally, it is less expensive and more available as a standard reagent than flunixin. Thus, in this work, we proposed a novel, simple, rapid, and reliable CE‐C^4^D method for determining FM by quantifying meglumine. The method was successfully applied for the determination of FM in veterinary pharmaceutical formulations.

## 2 Experimental Procedures

### 2.1 Reagents and Solutions

All reagents were of analytical grade. FM, lithium chloride, histidine (His), sodium hydroxide, 2‐(*N*‐morpholino)ethanesulfonic acid (MES), and *N*‐methylglucamine (meglumine) were purchased from Merck Sigma Aldrich (Darmstadt, Germany). Ultrapure water was obtained from a Direct‐Q3 water purification system (Millipore, Molsheim, France).

The BGE was prepared daily by dissolving proper masses of MES and His in ultrapure water to achieve a concentration of 20 mmol L^−1^ (each), resulting in a pH of 6.2. Stock solutions of FM, meglumine, and lithium chloride were prepared separately in ultrapure water at a concentration of 10 mg mL^−1^. The stock solutions of meglumine and FM were diluted with ultrapure water as required to prepare the working standard solutions and to spike the samples used in the recovery tests. To these solutions, a proper volume of the lithium stock solution was added as the internal standard (IS) to achieve a final concentration of 500 µg mL^−1^.

### 2.2 Instrumentation and Procedure

The CE‐C^4^D separations were performed using homemade equipment with a bare fused silica capillary column, 50 cm long (42 cm effective), with an outer diameter of 360 µm and an internal diameter of 50 µm. Sample injection was performed hydrodynamically for 5 s using a pressure of 10 kPa. C^4^D was operated at 560 kHz and 2 V (peak‐to‐peak). Capillary conditioning was performed daily by flushing with NaOH 0.1 mol L^−1^ (5 min), ultrapure water (5 min), and BGE (10 min).

The peak areas in the electropherograms were obtained by integration using the software OriginLab 8.1 (OriginLab Corporation, North Hampton, MA, USA), which was also used for linear regression analysis of the calibration curves. Additional data treatment was performed using Excel 2024 (Microsoft Corporation, Washington, USA).

### 2.3 Sample Preparation

Three samples of veterinary formulations containing FM as the active pharmaceutical ingredient were analyzed. The injectable formulations Flunixin (UCBVET, Brazil) and Flumax (JA Saúde Animal, Brazil), identified as Sample 1 and Sample 2, were labeled to contain 91.2 and 83 mg mL^−1^ of FM, respectively. The tablet formulation Flunixin (Chemitec, Brazil), identified as Sample 3, was labeled to contain 20 mg of flunixin per tablet, corresponding to 33.2 mg of FM per 300 mg tablet (111 µg mg^−1^).

For the injectable formulations, they were only 100‐fold diluted with ultrapure water, and the IS was added at a concentration of 500 µg mL^−1^. For the tablet formulations, three tablets were initially crushed into powder and homogenized using a mortar and pestle. In the following, a mass of 200 mg of the powder, corresponding to 22.2 mg of FM (based on the label claim), was suspended in about 15 mL of ultrapure water in a beaker and sonicated for 10 min. After that, the suspension was quantitatively transferred to a volumetric flask, the IS (500 µg mL^−1^) was added, and the volume was made up to 25 mL with ultrapure water. Finally, 1.5 mL of this suspension was filtered through a PVDF (polyvinylidene fluoride) membrane filter (0.22 µm) and placed in a polypropylene vial for injection into the CE‐C^4^D system.

### 2.4 Validation of the CE‐C^4^D Method

The CE‐C^4^D method was validated in accordance with the guidelines of the Brazilian Health Regulatory Agency (ANVISA) for analytical method validation (RDC no. 166/2017), including the assessment of linearity, limits of detection (LOD) and quantification (LOQ), precision, and accuracy. The calibration curve was obtained using meglumine standard solutions corresponding to FM concentrations of 100, 500, 1000, 1500, 2000, and 2500 µg mL^−1^, which were injected in triplicate. The calibration curve was based on the ratio of the meglumine peak area to that of the IS (Li^+^). To validate the use of meglumine as a standard reagent for the indirect determination of FM, another calibration curve was obtained using FM standard solutions at the same concentration levels, also injected in triplicate. For both curves, the IS concentration was kept constant at 500 µg mL^−1^. The LOD and LOQ were calculated on the basis of signal‐to‐noise ratios of 3 and 10, respectively. The accuracy of the method was evaluated by recovery tests conducted for each sample spiked with FM at three concentration levels. The spiked samples were prepared as described in Section 2.3. Intra‐ and inter‐day precisions were calculated for FM determinations conducted in triplicate on the same day and on 3 different days, respectively.

## 3 Results and Discussion

### 3.1 CE‐C^4^D Separation Optimization

**FIGURE 2:**
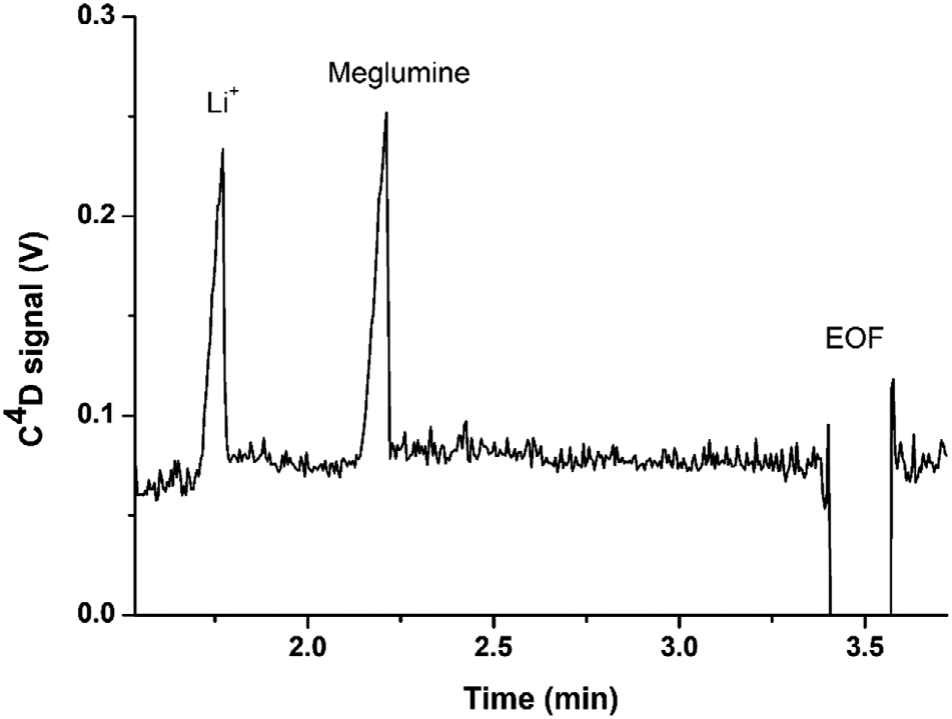
Electropherogram of a standard solution containing meglumine (400 µg mL^−1^) and Li^+^ (500 µg mL^−1^). EOF corresponds to the electroosmotic flow peak. BGE: MES/His 20 mmol L^−1^ (each), pH 6.2; bare fused silica capillary column with a total length of 50 cm (42 cm effective), 360 µm o.d., and 50 µm i.d.; separation voltage of 25 kV; hydrodynamic sample injection (5 s at 10 kPa); C^4^D operating at 560 kHz and 2 V (peak‐to‐peak). C^4^D, capacitively coupled contactless conductivity detection.

Meglumine has a pKa of around 9.5, so the MES/His (20 mmol L^−1^, each) buffer at pH 6.2 was evaluated as BGE because at this pH meglumine is protonated, allowing its separation as a cation. Additionally, this BGE composition has a low conductivity background, which is suitable for C^4^D. Figure 2 shows an electropherogram in which the meglumine peak is observed with a migration time of 2.2 min. The same electropherogram shows a peak for lithium ion at 1.77 min, which was chosen as the IS because it has a migration time close to that of meglumine and was absent in the analyzed samples. The intense negative peak at 3.5 min corresponds to the low‐conductivity injection zone, which migrates at the electroosmotic flow (EOF) velocity.

### 3.2 Analytical Parameters of the CE‐C^4^D Method

Table 1 summarizes the main analytical parameters evaluated for the proposed method. The relative standard deviation (RSD) of the migration time was below 1% for ten consecutive runs. The RSD was 1.4% for the migration time obtained on three different days, indicating good inter‐day precision. The RSD values for the peak area ranged from 4.5% (intraday) to 6.1% (inter‐day) and were higher than those typically observed in chromatographic methods (<1%). However, these precisions are in accordance with those usually reported for CE‐C^4^D methods, which are inherently more sensitive to variations in EOF and temperature.

**TABLE 1:** Main analytical parameters of the capillary electrophoresis with capacitively coupled contactless conductivity detection (CE‐C^4^D) method.

Parameter	
Migration time (min)^a^	2.21 ± 0.02
Intraday RSD migration time (%)^a^	0.9
Inter‐day RSD migration time (%)^a^	1.4
Intraday RSD peak area (%)^a^	4.5
Inter‐day RSD peak area (%)^a^	6.1
N m^−1^ ^b^	56 657
Dynamic linear range (µg mL^−1^)^c^	100–2500
Calibration curve equation^d^	*y* = (1.75 × 10^−3^)*x* − 0.12
Coefficient of determination (*R* ^2^)	0.992
LOD (µg mL^−1^)	22.7 (9.00)^e^
LOQ (µg mL^−1^)	75.6 (30.0)^e^

Abbreviations: LOD, limits of detection; LOQ, limits of quantification; RSD, relative standard deviation.

^a^ For meglumine peak (*n* = 10 intraday; *n* = 3 inter‐day).

^b^ N m^−1^ = 16 (*t*_m_/*w*_b_)^2^/*L*; *t*_m_, migration time; *w*_b_, peak base width; *L*, effective capillary length.

^c^ FM‐equivalent concentration.

^d^ *y* = ratio of the peak areas of meglumine and IS; *x* = FM‐equivalent concentrations in µg mL^−1^.

^e^ Concentration in parentheses for meglumine.

The usual high CE separation efficiency can be attested to by the high number of plates per meter achieved.

Table 2 compares the main parameters of the calibration curves obtained using meglumine and FM as standard reagents. Although the calibration curve obtained with FM showed slightly better linearity, the slope and intercept values were statistically similar at the 95% confidence level. This similarity indicates that meglumine can be used as an alternative, accessible, and lower cost standard reagent for FM determination, without loss of analytical performance. Nevertheless, this approach is only valid when FM is present, and there are no other sources of meglumine, as in the pharmaceutical and veterinary formulations.

**TABLE 2:** Comparison of parameters of the calibration curves of flunixin meglumine (FM) obtained using FM and meglumine standard solutions.

Parameter	Meglumine	FM	*t*‐test^a^
Linear dynamic range (µg mL^−1^)	100–2500^b^	100–2500	—
Slope	(1.75 ± 0.08) × 10^−3^	(1.62 ± 0.03) × 10^−3^	*t*_cal_ = 1.62 < *t*_crit_ (2.45)
Intercept	−0.12 ± 0.03	−0.27 ± 0.08	*t*_cal_ = 1.70 < *t*_crit_ (2.45)
*R* ^2^	0.992	0.999	—

^a^ Significance level of 95%.

^b^ Converted to equivalent FM concentration.

Regarding sensitivity, the LOD and LOQ were considered suitable for quantifying FM in the analyzed samples, considering the concentration levels typically found in pharmaceutical formulations.

As depicted in Table 3, the recovery ranged from 89% to 104% with a maximum standard deviation of 8%. These results suggest that the proposed method has appropriate accuracy for quantifying FM in veterinary formulations. Nevertheless, this indirect determination of FM assumes a 1:1 stoichiometric ratio between flunixin and meglumine. Deviation from this ratio, due to additional sources of meglumine, may affect the accuracy of the proposed method.

**TABLE 3:** Recovery values at three concentration levels of flunixin meglumine (FM) obtained by the capillary electrophoresis with capacitively coupled contactless conductivity detection (CE‐C^4^D) method.

Sample	Concentration added^a^	Concentration found^a^	Recovery ± SD (%)^b^
1 (Injectable)	25.2	26.2	104 ± 8
	50.4	44.3	89 ± 1
	75.6	73.3	97 ± 4
2 (Injectable)	25.2	23.1	93 ± 6
	50.4	52.4	104 ± 7
	75.6	76.4	101 ± 8
3 (Tablet)	31.5	35.5	103 ± 2
	63.0	60.0	95 ± 3
	93.2	97.0	104 ± 8

^a^ Concentrations before dilution; mg mL^−1^ for the injectable formulation and µg mg^−1^ for the tablet formulation.

^b^ Mean and standard deviation (*n* = 3).

### 3.3 Sample Analysis by CE‐C^4^D Method

**FIGURE 3:**
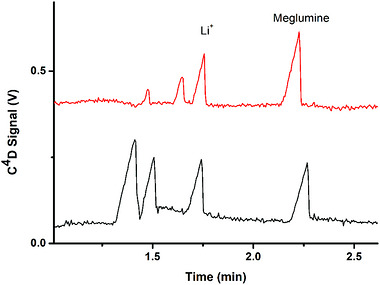
Electropherograms of a 100‐fold diluted injectable formulation (black line) and 200 mg of a tablet formulation (red line) extracted in 25 mL of ultrapure water. Separation conditions are as in Figure 2. C^4^D, capacitively coupled contactless conductivity detection.

Figure 3 shows electropherograms of an injectable and a tablet formulation after aqueous dilution and extraction, respectively. The peaks with migration times lower than the IS (Li^+^) are attributable to unidentified cationic compounds present as excipients in the formulation that were not disclosed on the label.

Table 4 displays the concentrations determined in the veterinary pharmaceutical formulations using the proposed method, which can be compared with those labeled by the manufacturers. The found concentrations ranged from approximately 98% to 108% of the labeled values. Although the manufacturer specification limits were unavailable, these values fall within or are close to ranges commonly accepted for pharmaceutical formulations (90%–110%). The higher FM concentration observed in the tablet formulation is unlikely to be due to matrix effect, as the recovery values for this sample were close to 100% at three concentration levels. In pharmaceutical formulations, concentrations of the active ingredient above those listed on the label may be related to manufacturing tolerances and stability considerations. For the sample analysis, the intraday RSD (*n* = 3) varied from 2.3% to 3.4%, whereas the inter‐day RSD (three different days) ranged from 3.1% to 4.2%. Usually, RSD values below 5% indicate acceptable precision for quantitative analysis.

**TABLE 4:** Flunixin meglumine (FM) concentrations in the veterinary formulations determined by the proposed capillary electrophoresis with capacitively coupled contactless conductivity detection (CE‐C^4^D) method and those informed by the manufacturers.

Sample	Label claim (manufacturer)^a^	Conc. found by the CE‐C^4^D^a^ ^,^ ^b^
1 (Injectable)	91.2	89 ± 3
2 (Injectable)	83	88 ± 2
3 (Tablet)	111	120 ± 3

^a^ mg mL^−1^ for the injectable formulation and µg mg^−1^ for the tablet formulation.

^b^ Mean and standard deviation (*n* = 3).

### 3.4 Comparison With Other Methods

Table 5 compares some analytical parameters of the proposed CE‐C^4^D with those reported in the literature for the determination of FM in pharmaceutical or veterinary formulations. All the compared methods used FM as a standard reagent, and most of them are based on HPLC separations, which usually require a larger volume of solvent. The linear dynamic range of the proposed method was comparable to or wider than that of the other methods. On the other hand, the LOD and LOQ were higher but were suitable for determining the FM concentrations typically found in veterinary formulations. Additionally, the method is simpler, faster, requires a lower cost instrument, and generates fewer residues than HPLC methods. The recovery values of the CE‐C^4^D method were close to those reported for the other methods, indicating good accuracy.

**TABLE 5:** Comparison of the capillary electrophoresis with capacitively coupled contactless conductivity detection (CE‐C^4^D) method with other methods for the determination of flunixin meglumine (FM) in formulations.

Technique	Samples	Linear range (µg mL^−1^)^a^	LOD (µg mL^−1^)^a^	LOQ (µg mL^−1^)^a^	Recovery (%)	Ref.
HPLC‐UV	Pharmaceutical formulations	302–970	—	—	94–100	[24]
Fluorescence	Injectable formulation	0.049–49	0.02	0.06	100	[13]
HPLC‐UV	Injectable formulation	43.8–175.4	0.5	1.7	99–101	[10]
HPLC‐UV	Injectable formulation	0.05–200	0.015	0.055	98–101	[5]
Micellar HPLC	Pharmaceutical formulations	0.1–20	0.02	0.06	96–107	[11]
CE‐C^4^D	Veterinary formulations	100–2500	22.7	75.6	89–104	This work

Abbreviations: HPLC, high‐performance liquid chromatography; LOD, limits of detection; LOQ, limits of quantification.

^a^ Concentrations expressed as flunixin meglumine.

## 4 Conclusions

FM was determined by quantifying meglumine, the counterion of flunixin, using CE‐C^4^D. This approach is advantageous because it allows the use of meglumine as a standard reagent, which is less expensive and more readily available than flunixin or FM. The CE capability for separating ionic and ionizable compounds and the C^4^D suitability for detecting compounds with low or no UV–vis absorption, such as meglumine, were fundamental to the proposed method. The CE‐C^4^D method has been demonstrated to provide rapid separation and is simple, reliable, and environmentally friendly. Thus, it can be considered a useful alternative for quality control and stability studies of veterinary pharmaceutical formulations containing FM. Moreover, the method can be used or adapted to determine meglumine in other matrices. However, as an indirect method, its applicability is limited to samples containing 1:1 FM salt.

## Conflicts of Interest

The authors declare no conflicts of interest.

## Data Availability

The data that support the findings of this study are openly available in the public repository of the State University of Campinas (REDU), https://redu.unicamp.br/.
